# Editorial and Miscellaneous

**Published:** 1862-04

**Authors:** 


### EDITORIAL AND MISCELLANEOUS.

*Meeting of the State Medical Society—Postponement of the Meeting of the Am. Med. Association.*—The attention of the profession is directed to the subjoined notices which have been enclosed to us for publication.

At the last meeting of the State Medical Society it was made the duty of the Secretary to call the meeting for this year in the month of May, contingent upon the assembling of the American Association at that time, otherwise to call it in the month of June.

The Committee of Arrangements of the National Association, having (as we conceive wisely) concluded that the causes which led the postponement of the annual meeting last year still operated, and with perhaps greater force, and therefore having determined upon still another postponement, the result is that the State Society will not meet until the time announced below.

The importance of bearing these changes in mind will be apparent to all who intend coming up to the annual gathering, which, it is to be anticipated, will exceed, both in interest and numbers, any previous anniversary of the Society.

Meanwhile we must be permitted to congratulate the members of the American organization that their Committee of Arrangements have not been tempted into a premature rally of the Association by the ill-advised urgency of a few individuals who seem even yet wholly unaware of the terrible struggle for mere existence in which the nation is now engaged. It is enough to say wiser councils have prevailed.

### ANNUAL MEETING OF THE AMERICAN MEDICAL ASSOCIATION.

We, the undersigned, Committee of Arrangements of the American Medical Association, after free consultation with officers and members in each important section of the country accessible to the Committee, feel constrained to give notice to the Profession, that the regular annual meeting of the Association is further postponed until the first Tuesday in June, 1863.

Chicago, March 20th, 1862.

N. S. Davis,
J. Bloodgood,
J. W. Freer,
DeLaskie Miller,
H. W. Jones,
E. Andrews,
Thos. Bevan,
} Committee.

### ELEVENTH ANNUAL MEETING OF THE ILLINOIS STATE MEDICAL SOCIETY.

The regular annual meeting of the Illinois State Medical Society will be held in Jacksonville, commencing on the first Tuesday in June, 1862. A full attendance of delegates and members from all parts of the State is very desirable.

Chicago, March 3rd, 1862.

N. S. Davis, Permanent Secretary.

---

*An Error Corrected.*—The *Lancet and Observer* errs in its statement that Rush Medical College did not continue its last session through the usual period. It falls into this error, possibly, from the fact that the last session *commenced* two weeks earlier, and of course closed two weeks earlier than previously had been the custom. The session continued uninterruptedly from the first day to the very last day of the usual period, and, we are happy to inform our contemporary, was attended by more than its usual average of students. Of which latter fact the forthcoming catalogue and announcement will be full evidence.

The change in the time of commencing the annual session was made at the solicitation of a large number of students, and has been found practically so convenient, that it will probably become permanent.

So far from abbreviating the lecture season, or wishing to do so, Rush Medical College stands ready to coincide in any well devised or general movement for increasing the length of terms. But be it observed, it does not believe in taking out of the body what is put on the extremities—spreading the same number of lectures over a greater space of time, thus encouraging indolence both in the teacher and the pupil. From that sort of reform it begs to be excused.

---

*Pyro-Phosphate of Iron.*—Several correspondents inquire where this preparation (an article concerning the merits of which was contained in the last number of this journal) can be procured of a reliable character. It may be that other parties have it, but we have been in the habit of ordering it from Mr. E. H. Sargent, corner of State and Randolph streets, in this city, who puts it up in most excellent style.

The article is an elegant one, and one which cannot fail to become popular among those who have an eye to æsthetics in medicine. It avoids the nauseous inky taste of the chalybeates generally, is agreeable to sight, taste and smell, and usually acceptable to the stomach. We say usually, for in a few instances it has seemed to provoke colicky pains and diarrhœa when given in full doses. This has once or twice necessitated the exhibition of aromatics, etc., and even temporary discontinuance of the medicine. Whether the phosphorus in its composition has the remarkable efficiency asserted for it by Dr. Chapman, we are inclined to doubt; but its chemical characteristics certainly suggest it for trial in many peculiarly intractable cases.

---

*Penetrating Wound of the Lung.*—Prof. E. C. Lane, in the *San Francisco Medical Press*, records a very interesting case of punctrred wound of the lung, the knife entering between the second and third rib, near the sternum on the left side. The rush of air in and out of the wound, and the gush of blood, are graphically portrayed. The successful treatment involved as its striking characteristic the careful removal of the extravasated blood from the pleural cavity *immediately*, and not as generally directed, closing up the wound and allowing it to remain till some days afterwards. He observes: "The rapid convalescence of the case, the escape from pulmonary inflammation, together with the avoidance of empyema—which would have been the event of the opposite course of treatment—would induce me to evacute, *instanter*, blood from the thoracic cavity, in all cases of wounds penetrating it, and accompanied by internal hemorrhage.

Germain to this point, we recall attention to the interesting cases of gunshot wounds of the lungs reported in this journal in February, 1861, by Dr. Clapp, now of California.

---

*A Treatise on Gunshot Wounds.*—By T. Longmore, Esq., Deputy Inspector-General of Hospitals; Professor of Military Surgery at Fort Pitt, Chatham. Philadelphia: J. B. Lippincott & Co., 1862. 132 pp.

This little *brochure* is one of the books which sum up in brief the results of extended and diffuse observations. Without any especial claim to novelty, it affords a valuable, clear and concise discussion of gunshot wounds in general, as modified in effect by the peculiarities of particular parts and also practical directions for treatment.

It will be found useful not only to the Army Surgeon, for which it appears to be specially designed, but also for students and practitioners in civil life.

---

*Braithwaite's Retrospect.—Part the Forty-Fourth.*—Notice of the appearance of this valuable reprint was accidentally omitted in the last number of the Journal. It fully sustains the previous high character of the series, and will be found rich in practical information. All the booksellers have it.

*Anatomy, Descriptive and Surgical.*—By Henry Gray, F. R. S., F. R. C. S. and Lecturer on Anatomy at St. George's Hospital Medical School. With Drawings by H. V. Carter, M. D., Demonstrator, etc. Dissections jointly by the author and Dr. Carter. Second American from the revised and enlarged London Edition, with 395 engravings on wood. Philadelphia: Blanchard & Lea, 1862. 816 pp.

With the previous edition of this magnificent work every anatomist is familiar. We are certain that we do injustice to no author when we say that it is pre-eminently *the* anatomical text-book. The general outline verges upon the absolutely perfect; particular descriptions are minute, lucid and exact, and the style throughout a classical model.

The engravings will arrest the attention of the most superficial observer for their boldness and strength; and they will command the admiration of the most critical for their perfection and beauty.

They have one peculiarity which conduces materially to convenience and perspicuity. The name of the part or point of interest is depicted immediately in its place on the engraving in such a manner, that while nothing is detracted from the perfection of the engraving, the student is not compelled to wade through foot-lines and a chaos of numbers or letters to find exactly the nomenclature of the parts.

Throughout the work, special attention is given to the surgical relations of every part, to such an extent, indeed, that the work is made indispensable to the practical surgeon. By the aid of the complete index, and the mode of indicating parts on the engravings, not a moment's time need be lost in referring to the anatomical and surgical relations to be studied. A brief account of the microscopical anatomy of some of the tissues and the various organs is also introduced.

In the present edition, inaccuracies have been corrected, "much additional matter has been added to the text, and several new illustrations executed with great care and fidelity by Dr. Westmacott, have been inserted."

The American edition has received the careful and judicious supervision of Dr. Richard J. Dunghican, and the name of Blanchard & Lea is sufficient guarantee of the typographical excellence of the volume. For sale by W. B. Keen & Co. and by S. C. Griggs & Co., Lake street.

---

*Characteristics of Parisian Notabilities.*—The most attractive lecturer at the college is certainly Malgaigne on operations and apparatuses. The speciality is admirably fitted for him as permitting of digressions into witticisms of the most bitter nature against inventors in general, and old Charriere in particular, who, by the way, is generally alongside. He is certainly the grand leveller. Few operations or instruments are better than blunders, his own excepted, and these invectives are uttered so eloquently, so beautifully sarcastic, that his hearers fail not to evince their appreciation in applause frequent and sincere. Many of the audience are non-professional, mere listeners indeed, who have no interest other than to hear the irony and watch the grimaces of this most peculiar speaker. He has such a crabbed appearance, and the contortion of his features as he is upon the point of saying something severe is so singular and unnatural, as to be comparable only to face of a snarling dog. A student ignorant of the language can tell when he bites. As a debater he is powerful and fearless, and in the meetings of the Academy of Medicine invariably puts down his antagonist.

Denonvilliers is perhaps the next most popular as a lecturer, and it must be by reason of his very oppositeness to his colleague, for no two men were ever more different than Denonvilliers and Malgaigne.

Jarjarvay makes the hour pass pleasantly, notwithstanding the difficulty of his subject.

From among the clinical professors Trousseau must be chosen as the true orator. Indeed this ought to be his forte, for in early life, besides being a legislator, he was professor of rhetoric. Few men are more admirable for both talent and exterior looks. In manner and appearance he reminds me greatly of Dr. Willard Parker.

Jobert de Lamballe is surgeon to the Emperor, and is besides as unfeeling as a Maisonneuve. He seems never pleased, for ever growling at his aids, doing everything but kicking them, and patients in his wards are scolded at like dogs.

What a contrast is the gentle Nelaton! All mildness with his assistants, and showing extreme sympathy for the sick. The largest *clientele* lies between him and Trousseau. I am told that Nelaton's income is about 200,000 francs.

Velpeau has been now so long walking the wards, and so long famous, that to foreigners in particular he has become a perfect curiosity. He is about the first one that the American student just arrived asks to be shown to. In a recent visit to Tours I understood that his studies were begun there, but so long ago that the oldest doctor did not remember. He has more internes and externes under his care than any other, especially of Spaniards. The crowd after him is so large that it is nearly impossible to see more than every fourth bed. He is familiar and kind to every one.

Piorry is called a great oddity with two great hobbies—the making of new sounds by his fingers, and new names with his tongue. For those who have faith to the very finest point in percussion he must be their king. As an instance of his perfection I may mention that I have known him to percuss the spleen some few hours after giving quinine, and detecting a diminution of its volume! He uses simply the pleximeter, to which he has given some new name which I forget. While percussing he never listens, at least scarcely ever. The *tactus eruditus* is upon his fingers, so that the slightest abnormality is at once perceived by them, and simultaneously, uninterruptedly, he announces to his followers the precise condition of the organ in question. His class is only moderate in size, and mostly made up of foreigners. I heard Maisonneuve once say that Piorry could detect and describe a clot beneath the cranium.

Bouillaud is no doubt a little vain of his resemblance to the first Napoleon. For my part I could never see it; but one thing is pretty certain, that is, although a physician he has spilled more blood than any surgeon in Paris. What does Bouillaud do? Bouillaud bleeds.—*Corr. Am. Med. Times.*

---

*Sore Nipples.*—Prof. Barker, in the *Medical Times*, without delaying to comment on the great variety of remedies proposed by the standard authors, makes a concise statement of what his experience has led him to believe the best method of treatment in each case.

*Erosion*,—Or when it is more extensive it is called *excoriation* of the nipple, is a superficial wound of the skin, in which the derm is laid bare by the removal of the epidermis by nursing. Sometimes it produces little vesicles, one or more, on the apex or sides of the nipple, which are broken by sucking, and the scales from which are again pulled off, and we have what the nurses call the *chapped* nipples. From this results entire destruction of the derm, and we then have *ulceration* of the nipple. The surface is then of a bright red color, granulated, and frequently swollen, and groved in fissures. When such a condition exists, you can readily understand that the act of nursing produces intolerable suffering, to such a degree that patients have often told me that the pains of labor could be more easily endured. I have sometimes seen half of the nipple bevelled off by this ulcerative process. But if you see the case sufficiently early, and treat it properly, and the nurse and patient scrupulously follow your directions, the ulcerative process may always be avoided. In the early stage of erosion and excoriation, direct that as soon as the child leaves the nipple it should be very carefully wiped dry, with a soft piece of linen, and then painted over by means of a camel hair brush, with the tinc. benzoin co. Brush it over three or four times, allowing an interval of a minute or two for each application to dry. This forms a kind of artificial cuticle, which should be renewed each time that the child nurses, and if it is possible to make the child nurse through it, direct that a nipple shield should always be used. Very good ones are now kept by our apothecaries generally, but in selecting one, be careful that its base is sufficiently large and elastic, so as not to strangulate the nipple. The first application of the benzoin produces a little smarting and burning pain for a moment or two, but its renewal is not usually painful. If the ulcerative process has commenced, stop nursing from the nipple. There is no other way, and the more promptly you decide to do this, the more speedily will the nipple be cured, and very frequently it is not necessary to suspend the nursing more than twenty-four or thirty-six hours. Empty the breasts by gentle rubbing only. This can only be done by tact and perseverance, although it sometimes requires ten minutes to get the first few drops. Then paint over the ulcerated surface, twice a day, with a solution of nitrate of silver, gr. x., ℥j. of distilled water, and keep the surface covered with carb. magnesia, or what I think is still better, calomel.

*Tissue or Crack*—At the base of the nipple occasions intense suffering, often I have thought quite as severe as the form of sore nipple that I have just described. It sometimes is so small that it can only be seen in a good light by bending the nipple over to the oppsite side. To cure it pencil the bottom of the fissure with a very fine point of the solid stick of nitrate of silver, and then cover it with collodion, that is the solution of gun cotton in sulphuric ether. If the fissure is not associated with the form of sore nipple that I have before described, or with inflammation of the nipple, that I am about to speak of, it is by these means cured speedily.

*Inflammation of the Nipple* is sometimes a cause and in other cases a consequence of the preceding conditions, and the inflammation frequently extends to the areola. The nipple is conical, red, swollen, and excessively painful. Apply a soft bread and milk poultice for a few hours, and then keep it covered with one or two thicknesses of linen, wet in a weak solution of leod water, as for example:—℞. Liq. plumbi diacet. dil. ℥j.; aq. rasæ ℥iij.; M. ft. lotio. After the inflammation is so far subdued that nursing can be borne without much pain, you will do well to substitute for the lead water the following:—℞. Aq. rosæ, glycerin., aa, ℥ij.; acidi tannic. ʒij.; M. ft. lotio. I have described each of the above forms of sore nipples as distinct affections, but you should not forget that they may be associated, either two forms or the three together, when the treatment must be modified or combined according to the special indications.

*Eczema of the Nipple*—Is, according to my experience, quite rarely met with, as I can only recollect six cases that I have seen. The first was at M. Velpeau's wards, at La Charite, in Paris. I have seen two cases in our lying-in wards, and three in consultation practice. Velpeau's prescription, which he said he had never known to fail, was the following ointment:—℞. Ung. aq. rosæ ℥j.; mag. carb. ℈ij.; hydrarg. chlor. nitr. ʒj. M. You should direct the apothecary to rub it up very thoroughly, or it will be lumpy. This ointment cured in a few days the cases we had in this hospital, but I am not able to say how successful it was in other cases.

---

*Hydrophobia.*—Dr. M. C. Hazen, of Haddam, Ct., reports in the *Boston Med. Jour.* a case of hydrophobia referred to the bite of a dog *eleven years previous.* The dog lived two years after inflicting the wound, and then died from a fall, breaking his neck. The same writer refers to a case in an adjoining town, where a man with hydrophobia (terminating fatally) bit two persons who were attending him, both of whom died of well-marked hydrophobia, the last fifteen years after.

Dr. Hazen's case had a remarkable inclination to snap and bite those around.

The treatment essentially consisted in moderately full doses of Morphiæ and Stramonium, a free mercurial purge the third day, a blister on the calf of the leg; the sixth day, small doses of Quina and Ar. Sulph. Acid. The patient recovered.

We are inclined to the opinion that the case was not one of hydrophobia. The details of convulsive action, inclination to bite, &c., so far as given, do not indicate more than the presence of some centric or excentric cause of irritation of the nervous system, which, unfortunately, was not discovered, because of the preconceived idea of hydrophobia, which had taken possession of the attendant's mind so thoroughly as to exclude search after the real lesion. The successful result of a case, or even appropriate treatment, does not by any means necessarily confirm a diagnosis. Particular groups of symptoms being, for convenience, adopted as designating particular diseases, it happens unluckily that sometimes diseases come to be looked upon as positive entities, as was evidently the fact in this case of so-called "hydrophobia."

Hydrophobia is a good deal like "worms" in popular pathology—it covers a great variety of affections. Real cases of hydrophobia are as rare as tetanus from any single cause; the pathological group of symptoms just about as common as tetanus from the variety of causes, idiopathic (undiscovered ?) or traumatic, which may give rise to it.

Of course the "barking and snapping and biting" every modern pathologist understands the meaning of.

---

*Uterine Hæmorrhage.*—Dr. Conkling (Med. Times) speaks highly of the introduction into the *os* of Liq. Ferri Per Sulph. by means of sponge or a strip of muslin, or by direct injection. The vagina should then be plugged thoroughly in the usual manner.

*Delirium Tremens.*—Dr. Lewis A. Sayre, in the same paper, reports successful use of the iced bath in the treatment of delirium tremens. The suggestion was made to him by Dr. Bauer, of Brooklyn. The case was immediately under the care of Dr. Orsamus Smith, of Blackwell's Island Hospital. The powerful sedative influence of the cold was well illustrated.

---

*Pirogoff's Operation.*—B. Frank Palmer, the man who, *Punch* insisted, makes better artificial legs than Nature does by her previously supposed inimitable processes, objects to Pirogoff's operation, as leaving the extremity little fitted for serviceable use, and at the same time badly adapted for supplying artificial compensation. He suggests for the surgeons' consideration in amputating the *leg* and *thigh* the following places of election:

1st place of election. The *lower third or fourth of the leg.* Flap operation. Remove the malleoli fully *always.*

2d. The lowest point possible between the first place of election and the upper third at which a good *flap* can be made.

3d. Immediately below the tuberosity of the fibula, if not practicable to save four inches below the patella, *with full use of joint.*

4th. The lower third of the thigh—*ten inches from perineum.* Always fully remove the condyles of the femur. Flap operation.

5th. *The utmost length possible,* if necessary to amputate above the fourth place of election. Flap operation.

These points, he states, have the approbation of the most eminent surgeons in this country and Europe.

---

*Quinine as a Prophylactic.*—A very sensible correspondent of the *Reporter* sustains views which we have heretofore advanced on this point as follows:

It has been a question with me if this Commission has not done more hurt than good, especially in the recommendation of quinine as a prophylactic for intermittents. I must question the propriety of giving quinine in a perfect state of health for the purpose of preventing an ill effect from malaria. Those cases that have been reported as being benefitted by the supposed prophylactic power of quinine, if theory or reason is good for anything, no doubt in such cases there existed an incipient form of disease—at least, were under the influence of malarial poison.

It was my privilege to spend a few weeks in our army—in Virginia, on Arlington Heights—last autumn. A number of cases of the form of disease that prevailed at that time came under my observation. Almost every case assumed, at first, an intermittent type of fever, attended with a congestive form of diarrhœa. I observed in the treatment quinine and opium were given liberally in combination—in many of these cases the fever assumed a continued form, with increased enteric symptoms. In this state of the disease they were removed from the army hospital tents to one of the hospitals in Washington or Georgetown, where they were treated for typhoid fever, as I was informed. A few cases were attended by myself. In all these cases I prescribed opium with hydrarg. c. creta, sinapisms over the abdomen until the tenderness upon pressure was relieved, after which quinine was used with marked benefit. One of these patients had a relapse before I left; the symptoms were much severer than in the first attack, ten days or so previous. The surgeon of the regiment remarked, in examining, at the *time of the relapse*, that it was frequently the case that his patients had a relapse, and *that* this was one of the ordinary type, and that he would now have a course of typhoid fever, and there could be no escape. I prescribed for him, as at first, large doses of opium combined with mercurial chalk, aided with counter-irritants over the abdomen, until the congestive form of disease was subdued, after thirty-six to forty-eight hours' duration. Quinine was then administered with the happiest effects. How much this Sanitary Report, in favor of the prophylactic effects of quinine, has contributed to the *doubtful* practice, to say the least, is not known. One thing is certain, so far as I could learn: our troops never had quinine given them unless on the sick list. The Sanitary Report would seem to recommend it for the well and healthy. I am fully convinced that a mild form of disease was converted into an acute form by the too early administration of quinine, after which they were treated at one of the hospitals in Washington or Georgetown for *typhoid fever*—a *polite and learned prognosis.* A quinine treatment was followed up, and *some* did live through it. How much the report emenating from the Sanitary Committee led to the abuse of this remedy, of course, is mere presumption to say. The duty of this Sanitary Commission falls naturally, it seems to the writer, on the Brigade Surgeon and the Commissary; if so, why not abolish the whole system as worse than useless? W. D. S.

